# Differential Expression of Long Non-Coding RNA (lncRNA) in Mediterranean Mussel (*Mytilus galloprovincialis*) Hemocytes under Immune Stimuli

**DOI:** 10.3390/genes12091393

**Published:** 2021-09-09

**Authors:** Patricia Pereiro, Rebeca Moreira, Beatriz Novoa, Antonio Figueras

**Affiliations:** Instituto de Investigaciones Marinas (IIM), Consejo Superior de Investigaciones Científicas (CSIC), Eduardo Cabello, 6, 36208 Vigo, Spain; patriciapereiro@iim.csic.es (P.P.); rebecamoreiravigo@gmail.com (R.M.); beatriznovoa@iim.csic.es (B.N.)

**Keywords:** lncRNAs, RNA-Seq, Mediterranean mussel, hemocytes, PAMPs, immune response

## Abstract

The Mediterranean mussel is one of the most economically relevant bivalve mollusk species in Europe and China. The absence of massive mortalities and their resistance to pathogens affecting other cultured bivalves has been under study in recent years. The transcriptome response of this species to different immune stimuli has been extensively studied, and even the complexity of its genome, which has recently been sequenced, has been suggested as one of the factors contributing to this resistance. However, studies concerning the non-coding RNA profiles remain practically unexplored—especially those corresponding to the lncRNAs. To the best of our knowledge, this is the second characterization and study of lncRNAs in this bivalve species. In this work, we identified the potential repertoire of lncRNAs expressed in mussel hemocytes, and using RNA-Seq we analyzed the lncRNA profile of mussel hemocytes stimulated in vitro with three different immune stimuli: LPS, poly I:C, and β-glucans. Compared to unstimulated hemocytes, LPS induced the highest modulation of lncRNAs, whereas poly I:C and β-glucans induced a similar discrete response. Based on the potential cis-regulatory activity of the lncRNAs, we identified the neighboring protein-coding genes of the regulated lncRNAs to estimate—at least partially—the processes in which they are implicated. After applying correlation analyses, it seems that—especially for LPS—the lncRNAs could participate in the regulation of gene expression, and substantially contribute to the immune response.

## 1. Introduction

The Mediterranean mussel (*Mytilus galloprovincialis*) is a marine bivalve mollusk species with a worldwide distribution, being present on every continent. This species is commercially exploited mainly by Mediterranean countries (especially in Spain), but also by China, which is currently the main global producer [1]. The high success of its production is due, among other things, to its resistance to environmentally adverse conditions [2], and its natural resistance to marine pathogens that cause severe mortality episodes in other cultured bivalves, such as oysters and clams [3,4,5].

Although, as invertebrates, mollusks lack adaptive immunity, their innate immune system—which involves the immune cells known as hemocytes and a wide variety of molecular effectors—constitutes a very efficient defense mechanism [3]. In the particular case of Mediterranean mussels, their rich and high expression of antimicrobial peptides (AMPs) compared to other bivalves is considered one of the main factors explaining the disease resistance of this species [6,7,8,9]. Additionally, these animals show a complex and diverse repertoire of immune-related receptors and effectors [10,11,12,13,14]. 

Together with the Pacific oyster (*Crassostrea gigas*) [15,16,17,18,19,20], *M. galloprovincialis* is probably one of the most studied mollusk species in terms of immune response [21,22,23,24,25,26]. However, although numerous efforts have been made to understand the transcriptomic profiles of Mediterranean mussels, how the long non-coding RNAs (lncRNAs) are modulated in this species has remained totally unexplored. The lncRNAs are a type of non-coding RNAs (ncRNAs) longer than 200 nucleotides, and are transcribed in the same way as mRNA [27]; they regulate the expression of adjacent genes (cis-acting regulation) or distally located genes (trans-acting regulation) through a variety of mechanisms: epigenetic regulation, activation of transcription factors, chromatin remodeling, promoter activation, transcription interference, etc. [27,28]. However, the activity of certain lncRNAs is mediated by their own transcription process rather than by the transcripts themselves, making the encoded lncRNA transcript unnecessary [28]. Due to these complex interactions, to their additive effects, and to their ability to enhance or repress gene expression, it is difficult to establish concrete functions for specific lncRNAs. Moreover, transcripts identified as lncRNAs can be the result of transcriptional noise and, therefore, have no function [28]. However, it is known that lncRNAs are modulated after different immune stimuli in both invertebrates [29,30,31] and vertebrates [32,33,34,35], and even the involvement of certain lncRNAs in the immune system has been demonstrated in both animal groups [36,37,38,39,40,41]. In mollusks, some works have reported the identification of lncRNAs under different experimental conditions using RNA-Seq analyses, and they have been linked to a variety of processes, such as larval development, immune response or shell formation and pigmentation [42]. However, to the best of our knowledge, the lncRNA repertoire in *M. galloprovincialis* has only recently been investigated in gills from mussels exposed to *Vibrio splendidus* [43], but has remained unexplored in the main mussel immune cells—the hemocytes—under any experimental conditions. 

In a previous work, we described the mRNA and microRNA (miRNA) profiles in *M. galloprovincialis* hemocytes exposed to three different pathogen-associated molecular patterns (PAMPs)—lipopolysaccharide (LPS), polyinosinic:polycytidylic acid (poly I:C), and β-glucans—compared to unstimulated hemocytes [26]. These PAMPs were selected to mimic three different types of microorganisms: bacteria, viruses, and fungi. However, the lncRNA profiles remained to be elucidated. By applying a bioinformatics pipeline, from the complete batch of contigs obtained in that previous work, those potentially corresponding to lncRNAs were selected, and RNA-Seq analyses were conducted. The results showed that the three stimuli were able to modulate the lncRNA profile of the mussel hemocytes, with LPS being the stimulus inducing the most powerful response. Since lncRNAs are likely to alter the expression of their adjacent genes, the functionality of the lncRNAs identified in *M. galloprovincialis* was predicted based on the function of their neighboring protein-coding genes (located 10 kb upstream and downstream of the lncRNAs). In the case of LPS in particular, a high parallelism between the genes modulated after challenge and the neighboring genes of the lncRNA regulated was observed. Moreover, correlation analyses between modulated lncRNAs and those adjacent genes also modulated by the PAMPs revealed a high probability of lncRNA–gene interaction. These results could suggest that lncRNAs substantially contribute to the modulation of the immune response in hemocytes after stimulation. 

## 2. Materials and Methods

### 2.1. Animals

Ripe *M. galloprovincialis* (8–10 cm in shell length) were obtained from a commercial shellfish farm (Vigo, Galicia, Spain) and maintained in open-circuit filtered seawater tanks at 15 °C with aeration. Mussels were fed daily with *Phaeodactylum tricornutum* and *Isochrysis galbana*. Prior to the experiments, the mussels were acclimatized to aquarium conditions for at least one week.

### 2.2. Experimental Design, RNA Isolation, and Illumina Sequencing

**Figure 1:**
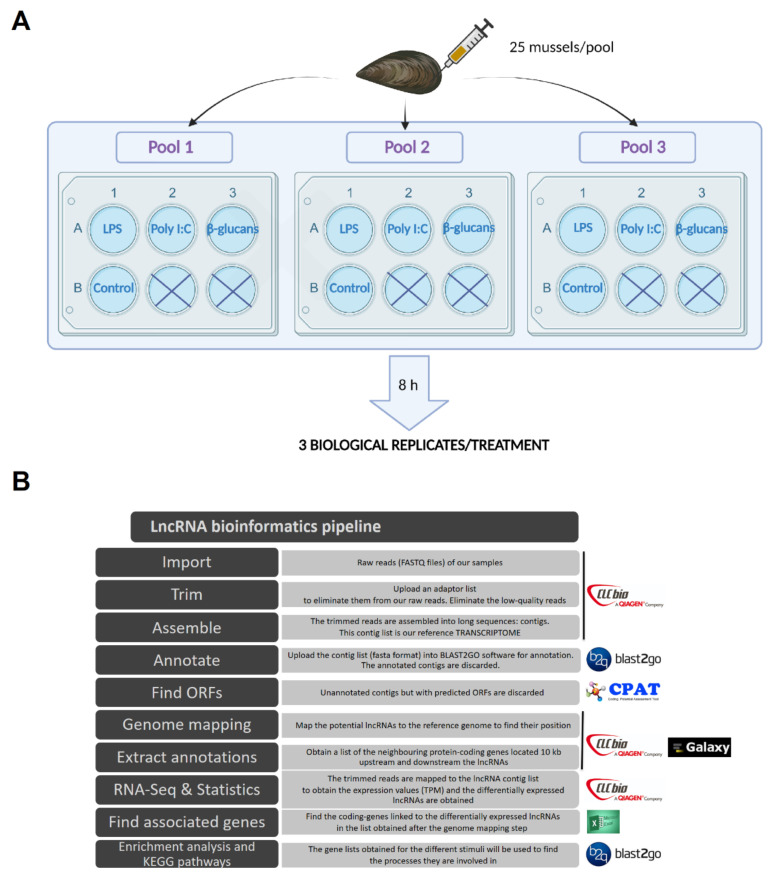
(**A**) Schematic representation of the experimental design and (**B**) bioinformatics pipeline conducted to analyze the *M. galloprovincialis* lncRNA profile.

As described previously [26], mussels were notched on the shell to gain access to the adductor muscle, and 1 mL of hemolymph was withdrawn from it with a 25 G disposable needle. Three pools containing the hemolymph from 25 individuals each were obtained. The hemolymph was distributed in 6-well plates, with 5 mL per well, in a total of 4 wells per pool. The hemocytes were maintained for 30 min at 15 °C in the dark and, after this period, they were stimulated for 8 h at 15 °C with 50 μg/mL of LPS, poly I:C, or β-glucans (one well per pool with each stimulus). The remaining well of each pool was not stimulated, and served as a control. Therefore, three biological replicates per treatment were obtained (a total of 12 samples). A schematic representation of the experimental design is shown in Figure 1A. All of the stimuli were purchased from Sigma-Aldrich (St. Louis, MO, USA). After the incubation with the stimuli, hemolymph was recovered from each well and centrifuged (4 °C, 3000× *g*, 10 min), and the pellets were used for RNA isolation with the Maxwell 16 LEV simplyRNA Tissue kit (Promega, Madison, WI, USA) using the automated Maxwell 16 Instrument, in accordance with instructions provided by the manufacturer. The concentration and purity of the isolated RNA was measured using a NanoDrop ND1000 spectrophotometer (NanoDrop Technologies Inc., Wilmington, DE, USA), and RNA integrity was analyzed using an Agilent 2100 Bioanalyzer (Agilent Technologies Inc., Santa Clara, CA, USA). Double-stranded cDNA libraries were constructed using the TruSeq RNA Sample Preparation Kit v2 (Illumina, San Diego, CA, USA), and sequencing was performed using Illumina HiSeq™ 4000 technology at Macrogen Inc. (Seoul, Korea). The raw reads from each sample were deposited in the Sequence Read Archive (SRA) (http://www.ncbi.nlm.nih.gov/sra (accessed on 31 December 2019)) with the following accession numbers: SAMN09104581 to SAMN09104592.

### 2.3. Trimming, Sequence Assembly, and lncRNA Mining

CLC Genomics Workbench v.12 (CLC Bio, Qiagen, Hilden, Germany) was used to filter, assemble, and perform RNA-Seq and statistical analyses. The pipeline reflecting the complete bioinformatics process is shown in Figure 1B. Raw reads were trimmed to remove low-quality reads (quality score limit 0.05) and adaptor sequences, and those sequences shorter than 70 base pairs (bp) were also trimmed out. A reference global transcriptome resulting from the 12 samples was assembled with an overlap criterion of 70% and a similarity of 0.9 to exclude paralogous sequence variants. The settings used were a mismatch cost = 2, deletion cost = 3, insert cost = 3, and minimum contig length = 200 base pairs. From the de novo assembly, the selection of the potential lncRNAs was conducted following a previously described pipeline [44], but with some modifications (Figure 1). Briefly, the generated contigs were annotated using BLASTx (*e*-value < 1 × 10^−3^) against the UniProt/SwissProt database, but also against a custom database composed of all of the molluscan sequences available in the NBCI nucleotide database. The annotated contigs were deleted from the assembly, as well as all of the contigs with an average coverage < 50. The potential open reading frames (ORFs) were predicted for the remaining contigs, and those with a potential ORF > 200 bp were discarded. After this, the Coding Potential Assessment Tool (CPAT) [45] was used to discard sequences with coding potential. The contigs that passed all of the filters were considered putative lncRNAs, and were retained for further analyses.

### 2.4. Genome Mapping and Identification of lncRNA-Neighboring Coding Genes

To position the putative lncRNAs on the genome, the *M. galloprovincialis* genome [46] was uploaded into the CLC Genomics Workbench v.12, and the putative lncRNAs were mapped against it. The obtained file (SAM format) was imported into the Galaxy web platform (https://usegalaxy.org/(accessed on 31 September 2020)) [47] and successively converted into an interval, BED and, finally, a GFF file, to be uploaded to the CLC Genomics Workbench v.12 for further annotation of the genome with the lncRNAs. By using the tool extract annotations, we obtained those protein-coding genes flanking up to 10 kb upstream and downstream from each mapped lncRNA. 

### 2.5. RNA-Seq and Differential Expression Analyses

RNA-Seq analyses were performed using CLC Genomics Workbench v.12 for each sample with the following parameters: length fraction = 0.8, similarity fraction = 0.8, and maximum hits per read = 10. The expression values were set as transcripts per million (TPM). A differential expression analysis test (Robinson and Smyth’s exact test, which assumes a negative binomial distribution of the data and takes into account the overdispersion caused by biological variability) was used to compare the expression levels between the samples obtained from the hemocytes stimulated with LPS, poly I:C, or β-glucans and the control samples, and to obtain the differentially expressed (DE) lncRNAs. Transcripts with absolute fold change (FC) values > 2 and a false discovery rate (FDR) < 0.05 were retained for further analyses. 

A heat map representing those lncRNAs that were differentially expressed with at least one of the PAMPs was constructed with the free software Heatmapper [48], using the mean TPM value for each experimental condition (mean of the three biological replicates). The distance metric was calculated using Pearson’s method, and lncRNAs were hierarchically clustered with the centroid linkage algorithm. Venn diagrams were constructed with the Venny 2.1 tool (http://bioinfogp.cnb.csic.es/tools/venny/(accessed on 31 July 2020)).

### 2.6. Gene Ontology (GO) Enrichment Analysis and KEGG Pathways

For the significantly DE lncRNAs in each comparison (LPS vs. control; poly I:C vs. control; β-glucans vs. control), the lncRNA-neighboring coding genes were extracted for GO enrichment analyses using Blast2GO software (BioBam, Valencia, Spain) [49]. Fisher’s exact test was carried out with default settings, a *p*-value cutoff of 0.05 was applied, and the enriched list was reduced to the most specific GO terms. The 20 most enriched biological processes (BPs) among the protein-coding genes proximal to lncRNAs were represented. 

The Kyoto Encyclopedia of Genes and Genomes (KEGG) pathways (http://www.genome.jp/kegg/pathway.html/ (accessed on 31 July 2020) in which the neighboring coding genes of the DE lncRNAs are involved were also obtained using Blast2GO software. 

### 2.7. Correlation Analyses between lncRNAs and Coding Genes

The expression levels of two pivotal immune gene families highly modulated by LPS were correlated with the expression of their DE neighboring lncRNAs. Correlations in the expression of both types of transcripts were calculated using Pearson’s correlation coefficient and Spearman’s rank correlation coefficient with IBM SPSS Statistics V25.0. To illustrate the expression correlations, heat maps were constructed with the free software Heatmapper [48], using the TPM values for each sample.

### 2.8. Quantitative PCR (qPCR) Validation of Differentially Expressed lncRNAs

A new experiment, but performed with individual mussels, was conducted following the same layout as the sequencing experimental design. Briefly, hemolymph from three individual mussels was extracted and divided among four wells (250 μL per well) in a 24-well plate. Three wells (one per mussel) were stimulated with poly I:C, β-glucans, or LPS, while the last served as a control. After 8 h, hemolymph was recovered from each well, and RNA was extracted as previously described. Three biological replicates per treatment were obtained.

cDNA synthesis of the samples was conducted using the NZY First-Strand cDNA Synthesis kit (NZYTech, Lisbon, Portugal) using 0.2 µg of total RNA. A total of 12 lncRNAs were used to validate the RNA-Seq results. Specific qPCR primers for the selected lncRNAs were designed using Primer 3 software (Free Software Foundation, Inc., Boston, MA, USA) [50], and their amplification efficiency was calculated via the threshold cycle (CT) slope method [51]. Primer sequences are listed in Supplementary Table S1. Individual qPCR reactions were carried out in a 25 µL reaction volume containing 12.5 µL of SYBR GREEN PCR Master Mix (Applied Biosystems, Foster City, CA, USA), 10.5 µL of ultrapure water, 0.5 µL of each specific primer (10 µM), and 1 µL of five-fold diluted cDNA template in MicroAmp optical 96-well reaction plates (Applied Biosystems). Reactions were conducted using technical triplicates in a 7300 Real-Time PCR System thermocycler (Applied Biosystems). qPCR conditions consisted of an initial denaturation step (95 °C, 10 min) followed by 40 cycles of a denaturation step (95 °C, 15 s) and one hybridization–elongation step (60 °C, 1 min). The relative expression level of the lncRNAs was normalized following the Pfaffl method [51] and using the *18S ribosomal RNA* (*18S*) as a reference gene, which was selected as a good reference gene for immune challenges based on previous analyses [52]. The correlation between the RNA-Seq and qPCR data was calculated using Pearson’s correlation coefficient, and by using the log10 of the FC.

## 3. Results

### 3.1. Assembly, Annotation, and lncRNA Mining

A summary of the reads, assembly data, and lncRNA mining is included in Table 1. High-throughput sequencing yielded an average of 71.4 million raw reads per sample, and an average of 98.71% of the raw reads per sample passed the quality control. After the de novo assembly step, a total of 219,765 contigs were obtained, with an average length of 493 bp. Of these contigs, 8.72% (19,168) were considered to be potential lncRNAs, which showed an average length of 500 bp. Most of these potential lncRNAs did not map against the *M. galloprovincialis* genome, and only 6234 (32.52%) lncRNAs were successfully positioned on the genome.

### 3.2. Differential Expression of lncRNAs after Stimulation of Hemocytes with PAMPs

**Figure 2:**
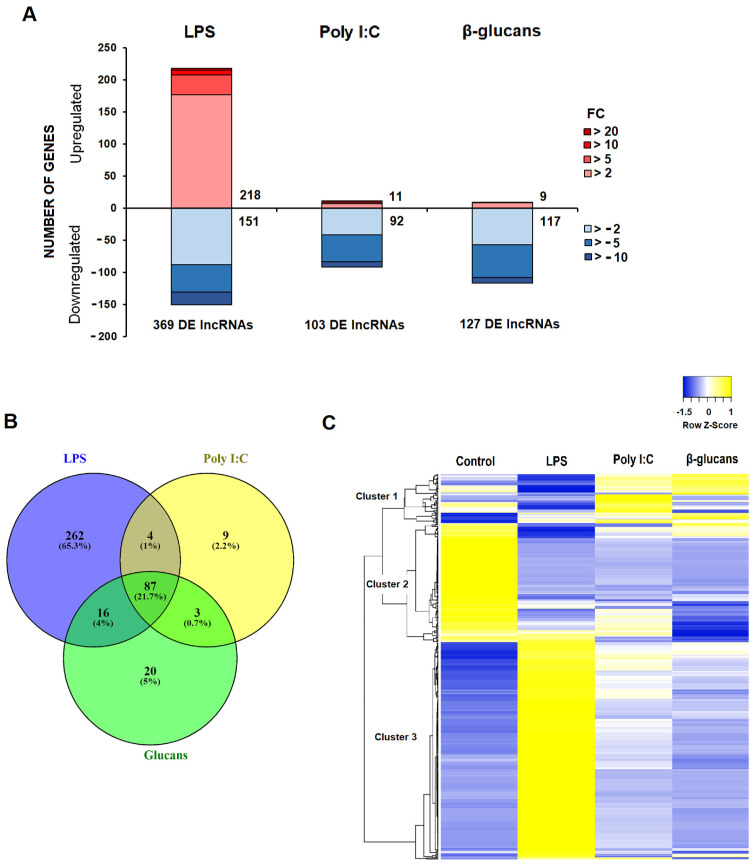
Modulation of mussel lncRNAs after the in vitro stimulation of hemocytes with three different PAMPs: (**A**) Stacked column charts reflecting the number of DE lncRNAs and the magnitude of the modulations induced by each stimulus compared to the unstimulated hemocytes. (**B**) Venn diagram reflecting the common and exclusive up- and downregulated lncRNAs significantly modulated by the different PAMPs. (**C**) Heat map reflecting the average TPM value for each DE lncRNA.

The differential expression analysis (FC > |2|, FDR < 0.05) for the PAMP-stimulated hemocytes compared to the control samples is provided in Supplementary Table S2. As is reflected in the stacked column charts (Figure 2A), LPS was the stimulus inducing the highest modulation of lncRNAs, with 369 DE lncRNAs (218 up- and 151 downregulated). On the other hand, poly I:C and β-glucans induced the modulation of 103 and 127 lncRNAs, respectively, most of them being downregulated (Figure 2A). This pattern observed for the lncRNAs was quite similar to that previously shown for the protein-coding transcripts [26].

A Venn diagram was constructed to illustrate the common and exclusive lncRNAs modulated in the mussel hemocytes by the different PAMPs (Figure 2B). Of the total lncRNAs that were differentially expressed after stimulation with PAMPs, most of them were exclusively modulated after LPS challenge (262 lncRNAs; 65.3%), followed by a pool of lncRNAs that were modulated by all three stimuli (87 lncRNAs; 21.7%). Those commonly modulated lncRNAs were affected in the same way (up- or downregulated) by the three stimuli. Only 20 (5%) and 9 (2.2%) lncRNAs were exclusively regulated by β-glucans and poly I:C, respectively (Figure 2B). When these lncRNAs were represented on a heat map, we observed three main clusters: one including those lncRNAs with a high expression after poly I:C and/or β-glucans stimulation (cluster 1); a second cluster grouping those lncRNAs whose mean TPM value was higher in the control samples (cluster 2); and a third, larger cluster containing the lncRNAs with the highest expression value in the LPS-stimulated hemocytes (Figure 2C).

### 3.3. Identification of the DE lncRNA-Neighboring Coding Genes

**Figure 3:**
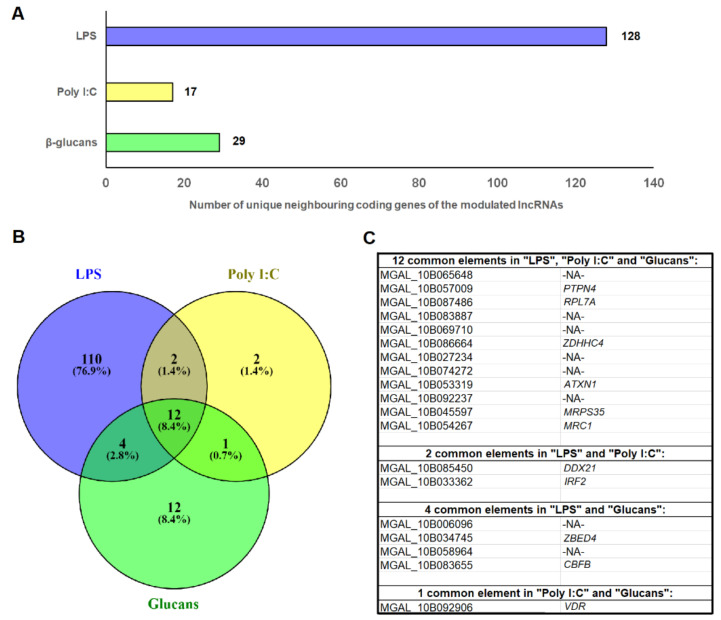
Identification of the neighboring coding genes of those lncRNAs differentially expressed in mussel hemocytes after immune stimulation. (**A**) Number of predicted genes in the *M. galloprovincialis* genome located in the 10 kb window of the DE lncRNAs. (**B**) Venn diagram showing the shared and exclusive genes located in the vicinity of the lncRNAs modulated by each PAMP. (**C**) Common genes located near lncRNAs significantly modulated by the different PAMPs. -NA- indicates non-annotated.

Although only 32.52% of the potential mussel lncRNAs were successfully mapped against the Mediterranean mussel genome, a total of 128 unique predicted genes were located on the 10 kb window around the lncRNAs modulated by LPS, whereas 17 and 29 were found for the lncRNAs regulated by poly I:C and β-glucans, respectively (Figure 3A; Supplementary Table S3). In some cases, more than one DE lncRNA surrounded one of these genes, which could suggest a strong regulation of these protein-coding genes by lncRNAs. Among these neighboring genes, 110 (76.9%) were exclusive to LPS, 2 (1.4%) to poly I:C, and 12 (8.4%) to β-glucans, whereas 12 (8.4%) were common to all three stimuli, and corresponded to commonly modulated lncRNAs (Figure 3B). Among the 12 common genes for the 3 PAMPs, half of them showed an informative annotation, corresponding to the following genes: *tyrosine-protein phosphatase non-receptor type 4* (*PTPN4*), *60S ribosomal protein L7a* (*RPL7a*), *putative ZDHHC-type palmitoyltransferase 4* (*ZDHHC4*), *ataxin-1* (*ATXN1*), *28S ribosomal protein S35, mitochondrial* (*MRPS35*), and *macrophage mannose receptor 1* (*MRC1*) (Figure 3C). Poly I:C and LPS shared two immunity-related genes: *ATP-dependent RNA helicase DDX21* (*DDX21*), and *interferon regulatory factor 2* (*IRF2*); LPS and β-glucans shared four common genes, two of them with annotation: *zinc finger BED domain-containing protein 4* (*ZBED4*), and *core-binding factor subunit β* (*CBFB*); finally, Poly I:C and β-glucans shared only one gene, annotated as *vitamin D3 receptor* (*VDR*) (Figure 3C).

### 3.4. GO Enrichment and KEGG Pathway Analyses of the lncRNA-Neighboring Coding Genes

**Figure 4:**
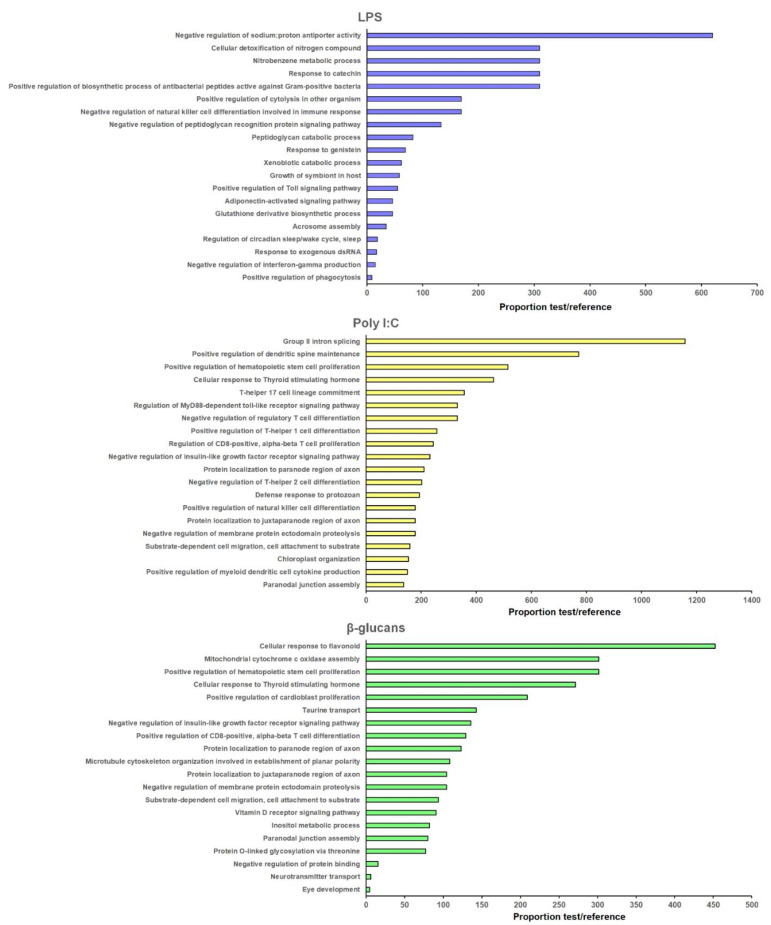
GO enrichment analyses of the neighboring coding genes of those lncRNAs differentially expressed in stimulated hemocytes. The 20 most enriched biological processes among the protein-coding genes proximal to lncRNAs are represented.

**Figure 5:**
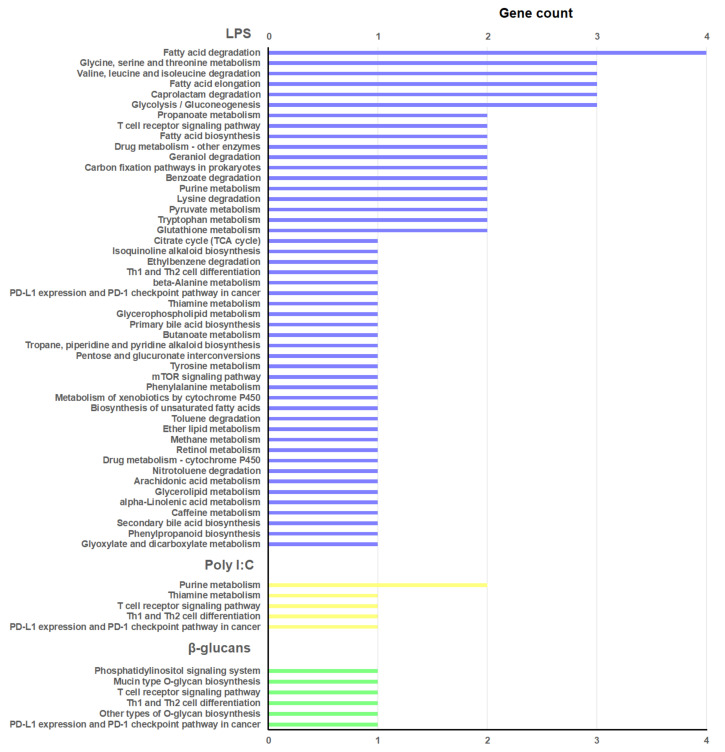
KEGG pathway analyses of the DE lncRNA-neighboring coding genes.

The protein-coding genes flanking the DE lncRNAs (Supplementary Table S3) were extracted for GO enrichment analysis. The 20 most significantly enriched BPs for each PAMP are represented in Figure 4, and the KEGG pathways in Figure 5. The three stimuli included BP terms and KEGG pathways related to the immune response.

For LPS, the enriched BPs were mainly related to the lncRNAs located in the vicinity of different genes annotated as peptidoglycan recognition proteins (PGRPs): “Positive regulation of biosynthetic process of antibacterial peptides active against Gram-positive bacteria”, “Positive regulation of cytolysis in other organism”, “Negative regulation of natural killer cell differentiation involved in immune response”, “Negative regulation of peptidoglycan recognition protein signaling pathway”, “Peptidoglycan catabolic process”, “Positive regulation of Toll signaling pathway”, “response to exogenous dsRNA”, “Negative regulation of interferon-γ production”, and “Positive regulation of phagocytosis” (Figure 4). Interestingly, four terms related to the detoxification of nitrogen and oxygen radicals were also enriched for the neighboring coding genes of those lncRNAs modulated by LPS, and are directly related to the glutathione S-transferase (GST) genes “Cellular detoxification of nitrogen compound”, “Nitrobenzene metabolic process”, “Response to catechin”, and “Glutathione derivative biosynthetic process” (Figure 4). A high representation of metabolic processes was observed among the KEGG pathways represented for these lncRNA-neighboring coding genes—especially those linked to the fatty acid metabolism (Figure 5). Some immune-related pathways were also observed (“T cell receptor signaling pathway”, “Th1 and Th2 cell differentiation”, and “mTOR signaling pathway”), as well as those related to cellular detoxification (“Drug metabolism—other enzymes”, “Glutathione metabolism”, “Metabolism of xenobiotics by cytochrome P450”, and “Drug metabolism—cytochrome P450”) (Figure 5).

For poly I:C, several immune-related BPs were also enriched (Figure 4)—especially those related to the T cells (“Positive regulation of hematopoietic stem cell proliferation”, “T-helper 17 cell lineage commitment”, “Regulation of MyD88-dependent toll-like receptor signaling pathway”, “Negative regulation of regulatory T cell differentiation”, “Positive regulation of T-helper 1 cell differentiation”, “Regulation of CD8-positive, α-β T cell proliferation”, “Negative regulation of T-helper 2 cell differentiation”, “Defense response to protozoan”, “Positive regulation of natural killer cell differentiation”, “Positive regulation of myeloid dendritic cell cytokine production”). These terms were directly related to the lncRNA-neighboring coding genes *ATP-dependent RNA helicase DDX21* (*DDX21*), *interferon regulatory factor 2* (*IRF2*), and *macrophage mannose receptor 1* (*MRC1*). Indeed, the two immune KEGG pathways represented for this PAMP were also linked to the T cells (“T cell receptor signaling pathway” and “Th1 and Th2 cell differentiation”) (Figure 5).

Finally, β-glucans was the stimulus with the lowest enrichment in immune BP terms (Figure 4): “Positive regulation of hematopoietic stem cell proliferation” and “Positive regulation of CD8-positive, α-β T cell differentiation”. As for poly I:C, the KEGG pathways “T cell receptor signaling pathway” and “Th1 and Th2 cell differentiation” were also represented (Figure 5). It is interesting to highlight that, for the neighboring coding genes of those lncRNAs differentially expressed in hemocytes after β-glucans challenge, some enriched BP terms and KEGG pathways related to the glycosylation process were observed. Among the enriched terms, we found the BP “Protein O-linked glycosylation via threonine” (Figure 4), and among the KEGG pathways we found “Mucin type O-glycan biosynthesis” and “Other types of O-glycan biosynthesis” (Figure 5); these were directly linked to the gene *N-acetylgalactosaminyltransferase 7* (*GALNT7*).

### 3.5. Correlation Analysis between Modulated Genes and lncRNAs

**Figure 6:**
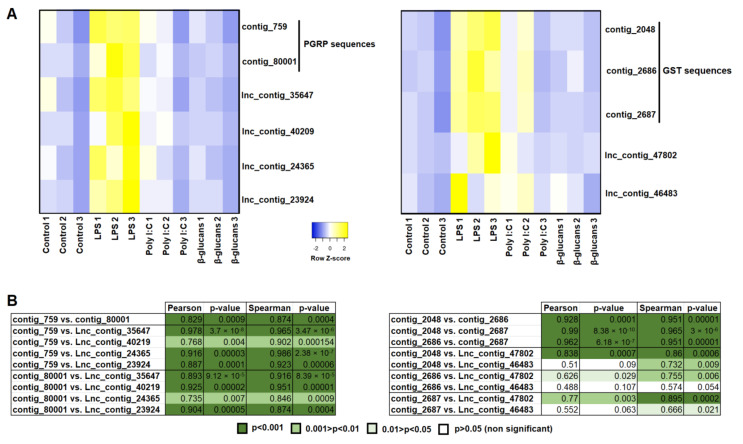
Correlation analyses of the DE lncRNAs and their neighboring coding genes. Those lncRNAs significantly modulated by LPS and located in the proximity of genes encoding for peptidoglycan recognition proteins (PGRPs) and glutathione S-transferases (GSTs), and the protein-coding contigs annotated as PGRPs and GSTs that were significantly induced by LPS, were selected. (**A**) Heat maps representing the TPM values of the lncRNAs and the protein-coding contigs per sample. Expression levels are represented as row-normalized values on a red–green color scale. (**B**) Correlation analysis between the lncRNAs and the protein-coding contigs. Pearson’s correlation coefficient and Spearman’s rank correlation coefficient were calculated, and their statistical significance (*p*-value) was considered.

To illustrate and analyze the correlation between PAMP-modulated genes and PAMP-modulated lncRNAs, we selected two groups of genes induced in hemocytes after LPS challenge [26], and with modulated lncRNAs located in the vicinity of genes belonging to those groups: peptidoglycan recognition protein (PGRP) genes, and glutathione S-transferase (GST) genes. The mean TPM values of the contigs encoding for PGRPs of GSTs and the corresponding lncRNAs were represented as heat maps, and the correlation between the protein-coding contigs and each neighboring lncRNA was calculated using Pearson’s and Spearman’s coefficients (Figure 6). In both cases, a strong and significant correlation between the protein-coding contigs was observed, and even between each contig and the different lncRNAs for the PGRP sequences. However, not all of the DE lncRNAs located near the GST genes showed a significant correlation with the DE contigs encoding for GSTs, although certain discrepancies were observed depending on the correlation method used (Figure 6).

### 3.6. Validation of lncRNA Expression Profiles by qPCR

To validate the RNA-Seq analysis of the mussel lncRNAs, we selected 12 lncRNAs DE in hemocytes with the different PAMPs (4 with LPS, 4 with poly I:C, and 4 with β-glucans). qPCRs were conducted in a different but equivalent experiment to that used for sequencing, serving as a technical and biological validation. Because individual mussels were used to validate the RNA-Seq results, and due to the previously demonstrated high individual variability in the gene expression levels of this species [8,11,24,46], some of the lncRNAs significantly modulated after PAMP challenges in the RNA-Seq experiment were not significantly modulated in the qPCR assay. Nevertheless, the tendency observed was the same for both experiments and techniques, with all of the lncRNAs being modulated in the same way (Supplementary Figure S1). In general terms, the magnitude of the FC was higher for the RNA-Seq data (Supplementary Figure S1A). A Pearson’s correlation analysis of the log10 FC revealed a high correlation between both datasets (r = 0.927) (Supplementary Figure S1B).

## 4. Discussion

Although the transcriptome response of Mediterranean mussels and other economically relevant mollusk species to different stimuli and/or conditions has been extensively studied, the non-coding RNAs profiles have remained practically unexplored until the past decade. However, miRNAs have been described and/or analyzed in a few mollusk species during the past decade [53,54,55,56,57,58,59], including *M. galloprovincialis* [26,60]. On the other hand, lncRNAs have been less studied, but some recent publications have reported the identification, expression profiles, and even the potential functions of some mollusk lncRNAs [29,61,62,63,64,65,66]. However, the identification and RNA-Seq analysis of *M. galloprovincialis* lncRNAs is limited to a recent publication describing the lncRNAs modulated in gills after *V. splendidus* bath infection [43].

The transcriptome of Mediterranean mussel hemocytes was sequenced under naïve conditions or after stimulation with three different PAMPs: LPS (bacterial stimulus), poly I:C (viral stimulus), and β-glucans (fungal stimulus). After Illumina sequencing, read trimming, and de novo assembly, a total of 219,765 contigs were obtained [26]. Following a pre-established filtering pipeline, a total of 19,168 contigs were selected as potential lncRNAs. This high quantity of lncRNAs contrasts with the more modest number of lncRNAs identified in gills, where only 6021 potential lncRNAs were determined following the same pipeline [43]. This fact could indicate that hemocytes express a large repertoire of lncRNAs compared to other tissues, playing an important role in their function and regulation. Indeed, although certain lncRNAs are ubiquitously expressed, many of them show tissue-specific expression patterns, even being restricted to a single cell type [67].

In a previous work, we analyzed the transcriptome and miRNome responses of mussel hemocytes after stimulation with these three immune stimuli mimicking three types of pathogens [26]. We found that LPS was the stimulus inducing the strongest immune response, although poly I:C showed a higher modulation of the miRNA profile. In this work, we analyzed the lncRNA profiles in these same samples. LPS was the PAMP inducing the highest modulation in the lncRNA repertoire, whereas poly I:C and β-glucans showed very discrete responses, and most of the DE lncRNAs were downregulated compared to the control hemocytes. Moreover, 21.7% of the lncRNAs that were significantly affected by at least one of the PAMPs were commonly modulated by all three stimuli, reflecting a low specific response for poly I:C and β-glucans. On the other hand, 65.3% of the DE lncRNAs were exclusively affected by LPS. This strong response induced by a challenge with LPS could indicate that Mediterranean mussels are highly efficient in the defense against bacterial pathogens compared to other types of microorganisms. However, due to the absence of widespread pathogen-associated mortality in this species, it is difficult to establish levels of sensitivity to different microorganisms.

As was mentioned in the introduction, lncRNAs can modulate gene expression through a variety of mechanisms, and these effects can result in either activation or inhibition of gene expression [27]. Therefore, lncRNAs are considered to be transcriptional units that contribute to the fine-tuned spatial and temporal gene expression programs [68]. At the same time, although the functional classification of lncRNAs remains very challenging, these transcripts can be divided into two groups based on the location at which the lncRNA acts: trans-acting and cis-acting lncRNAs [27,68]. Whereas trans-acting lncRNAs are transcribed, processed, and then migrate to distal locations to exert their function, cis-acting lncRNAs regulate the expression of neighboring genes, and these cis-acting lncRNAs constitute a substantial proportion of lncRNAs with an assigned function [66]. Therefore, in the absence of experimental evidence, the processes affected by an array of lncRNAs modulated under certain conditions could be at least partially estimated based on the function of their neighboring genes. Moreover, the rapid evolution of the lncRNAs and the consequent absence of conserved homologs for most of the lncRNAs—even in evolutionarily close species [69,70]—makes the identification of their functions difficult.

Due to the recent publication of the *M. galloprovincialis* genome [46], we were able to map the lncRNAs to the genome and obtain their positions. However, likely as a consequence of the complexity of the *M. galloprovincialis* genome, only 6234 (32.52%) of the 19,168 predicted lncRNAs were successfully mapped on the genome, which is quite similar to the result obtained for the lncRNAs identified in mussel gills, where 35% of the potential lncRNAs were mapped on the genome [43].

Based on their positions, we obtained information on the protein-coding genes flanking up to 10 kb upstream and downstream from the mapped and differentially expressed lncRNAs. These lncRNA-neighboring genes were used for GO enrichment analysis and prediction of the KEGG pathways in which they are involved. Interestingly, for LPS, we found a high parallelism between the BP GO terms enriched for the lncRNA-neighboring coding genes, and those enriched for the mRNA transcripts modulated by LPS [26]. Indeed, the GO enrichment analysis of the lncRNA-neighboring genes was dominated by terms related to cellular detoxification and/or oxidative stress, and the immune response was mainly linked to the PGRPs, as also occurred with the mRNA transcripts DE by LPS [26], with some of these terms being exactly the same. These results could suggest that the gene response of mussel hemocytes to LPS is highly controlled by cis-acting lncRNAs. With regard to the KEGG pathways, in both cases there was a strong representation of those related to the lipid metabolism, which is pivotal in the activation, differentiation, and function of immune cells [71]. Of course, this information should be interpreted carefully, since further functional analyses would be needed to confirm the interaction of the different lncRNA–gene pairs.

For poly I:C and β-glucans, these high similitudes were not observed, which could be influenced by the low number of BP terms enriched for the DE mRNA transcripts [26]. However, in this work, we observed that those lncRNAs modulated by poly I:C and β-glucans could be regulating several immune processes mainly linked to the T-cell proliferation and differentiation processes, as was also observed in the corresponding KEGG pathways. Although mollusks, as invertebrate animals, do not possess T cells, these terms reflect the enrichment of the lncRNA-neighboring genes in immune functions. It is also interesting to highlight the GO-enriched term “Protein O-linked glycosylation via threonine”, and the KEGG pathways “Mucin type O-glycan biosynthesis” and “Other types of O-glycan biosynthesis” observed for the neighboring genes of those lncRNAs affected by β-glucans. These are linked to the gene *N-acetylgalactosaminyltransferase 7* (*GALNT7*)—a member of the GalNAc-transferase family that controls the initiation step of mucin-type O-liked protein glycosylation by transferring N-acetylgalactosamine to serine and threonine amino acid residues [72]. Whether or not this enzyme is affected by the presence of β-glucans remains to be elucidated but, because β-glucans are polysaccharides consisting of glucose residues, and glucose can be transformed into galactose—and galactose into N-acetylgalactosamine—we cannot dismiss the potential effects of β-glucans on protein glycosylation.

To better elucidate the potential relationship between the DE lncRNAs and the protein-coding transcripts modulated by the PAMPs and transcribed by genes located in the proximity of these lncRNAs, we conducted a correlation analysis between both types of transcript. For this, we selected those lncRNAs significantly modulated by LPS and located in the proximity of genes encoding for PGRPs and GSPs,—two gene families highly affected by this PAMP—as previously shown [26]. The results revealed that all of the analyzed lncRNAs located in the vicinity of PGRPs showed a high positive and significant correlation with the contigs encoding for these proteins. Therefore, this gene family seems to be closely controlled by lncRNAs. On the other hand, not all of the DE lncRNAs located near GSP genes showed a significant correlation with the GSP transcripts, and one of them—lnc_contig_47802—seems to be the most strongly linked to the GSPs’ expression. 

## 5. Conclusions 

Although further functional assays would be needed in order to confirm these observations, these preliminary results provide interesting clues about the modulatory properties of lncRNAs in the main immune cells of mussels.

## Figures and Tables

**Table 1:** Summary of the Illumina sequencing, assembly, and lncRNA identification.

READS	
Total reads	856,863,196
Mean reads per sample	71,405,266
Mean trimmed reads	98.71%
**ASSEMBLY**	
Contigs	219,765
Minimum length	200 bp
Maximum length	16,164 bp
Average length	493 bp
N50	539 bp
**LncRNAs**	
Potential lncRNAs	19,168 (8.72%)
Average length	499.54 bp
lncRNAs mapped on genome	6234 (32.52%)

## Data Availability

The raw reads from each sample were deposited in the Sequence Read Archive (SRA) (http://www.ncbi.nlm.nih.gov/sra; accessed on 31 December 2019) with the following accession numbers: SAMN09104581 to SAMN09104592.

## Supplementary Materials

The following are available online at https://www.mdpi.com/article/10.3390/genes12091393/s1: Table S1: Primer pairs used to validate the lncRNA differential expression analysis; Table S2: Differential expression of the mussel lncRNAs in hemocytes stimulated in vitro with LPS, poly I:C, and β-glucans; Table S3: Predicted neighboring coding genes of the potential Mediterranean mussel lncRNAs; Figure S1: Validation of differentially expressed lncRNAs through qPCR.
